# A cross-sequential study of academic readiness and coping strategies among first-generation college students

**DOI:** 10.3389/fpsyg.2025.1537850

**Published:** 2025-03-05

**Authors:** Nimra Rani Musawar, Najia Zulfiqar

**Affiliations:** Department of Psychology, The University of Haripur, KPK, Pakistan

**Keywords:** academic readiness, continuing generation college students, coping strategies, first-generation college students, parental education

## Abstract

The transition from high school to college has embedded challenges, particularly for first-generation students. The study examined the overtime relationship and level of academic readiness and coping strategies among first-year, first-generation, and continuing-generation college students. Another objective was to examine the gender differences in the study variable. A cross-sequential design was used to collect data during college entry and 3 months after the baseline assessment. The differences in the levels of readiness and coping were examined based on participants’ generation status and gender. Generation status played a significant role in shaping readiness and coping strategies, and this association was more vital for the continuing generation than for the first-generation college students. As hypothesized, the findings show that first-generation college students were less ready and used poor coping strategies than continuing-generation college students at Time 1. However, this difference disappeared 3 months later between the two cohorts. The overall scores of readiness and coping increased from Time 1 to Time 2. Gender comparison showed that irrespective of being FGCS or CGCS, girls were more prepared than boys at the time of entering college, and boys surpassed girls in using coping strategies to overcome academic issues. Limitations, implications, and recommendations are discussed.

## Introduction

A first-generation college student (FGCS) is someone whose parents did not receive a bachelor’s degree or who did not attend their university (Ward et al., 2012). Peralta and Klonowski (2017) found several definitions of first-generation students in the literature review; these definitions refer to students whose parents never attended college and whose parents took some college courses but did not finish their degrees. A continuing generation college student (CGCS) has at least one bachelor’s degree or higher degree holder parent (Redford and Hoyer, 2017). In this study, we will use the abbreviated terms FGCS and CGCS to refer to first-generation and continuing-generation college students, respectively.

College readiness refers to “the preparation required to enroll in college and persist to graduation without need for remediation” (Duncheon, 2015, p. 15). Duncheon narrowed college readiness into three categories: (1) cognitive academic factors (i.e., content knowledge, cognitive skills); (2) non-cognitive academic factors (i.e., mindsets, behaviors); and (3) college knowledge (i.e., cultural and behavioral norms of higher education; relationship to self and others). Scholars developed college readiness models to assist educators in pursuing students’ excellence. Armstrong (2012) ecological academic preparation model considers the complexity of the individual and contextual variations and communication, organizational, and societal factors. This approach is based on racial, linguistic, socioeconomic, and other social and cultural markers. According to Arnold et al. (2012, p. 91), academic readiness refers to “the multidimensional set of skills, traits, habits, and knowledge that students need to enter college with the capacity to succeed once they are enrolled.” The “university-ready” refers to the ability of a college or high school student to pursue a graduate or higher degree.

Scholars have used high school grades and SAT/ACT scores to predict college students’ academic success. Combs et al. (2010) examined high-school students’ college readiness for reading, math, and both subjects. They reported that less than one-third of 1,099 students were considered college-ready in both subjects. The concept of academic readiness among FGCSs is of significant interest because they are under-resourced and lack formal guidance from home and family to adjust to the college environment. Research found that generation status significantly predicted academic readiness for SAT and ACT takers. FGCSs had lower scores on standardized tests and academic readiness than non-FGCSs (Balemian and Feng, 2013). Based on a longitudinal study, Conley et al. (2020) reported that an academically ready student may possess skills like goal setting, problem-solving, time management, analytical thinking, teamwork, and networking that FGCS lacks. Overall, college students have poor adjustment during the first 2 years, which improved during the third and fourth years of college. Most of the students had low levels of active emotional coping, social support, and self-esteem, but high levels of stress, anxiety, depression, and avoidant emotional coping (Conley et al., 2020).

FGCSs enter college academically less prepared than their peers who completed secondary education (high school, grades 9–12) and lack family support and necessary resources for college success (LeBouef and Dworkin, 2021). A study conducted in Pakistan by Ahmad et al. (2022) examined the relationship of FGCS with university adjustment. It showed that students come to college with high goals and motivation, but their first obstacle is adjusting to the academic environment. FGCSs are underprepared and unprepared to enroll in universities and to meet the curricular and co-curricular requirements because of family and work obligations outside the college. Students’ university preparation directly correlates with and influences their ability to transition to college (Ahmad et al., 2022). Crowell (2023) found that FGCSs more often exhibit lower levels of college readiness and reduced college enrollment and retention rates than CGCSs because of challenges related to finances, family support, work-life balance, and other issues. Despite these challenges, FGCS can benefit from college orientation programs, preparatory classes, family support, and institutional support. They must understand the challenges of higher education at entry into any institute and how to adjust to the environment of a university (Crowell, 2023). In light of the above literature, it is hypothesized that FGCS will have a lower academic readiness than CGCS.

Coping is an assessment process created to address internal and external obstacles. Coping strategies are actions used to control feelings, actions, thoughts, and environmental factors in reaction to the stress of daily living. Lazarus and Folkman (1984, p. 141) defined coping as “constantly changing cognitive and behavioral efforts to manage specific external and internal demands that are appraised as taxing or exceeding the resources of the person.” Lazarus and Folkman’s widely accepted transactional model considered stress as an interaction process between a person and their environment. This model has led to understanding coping as the cognitive and behavioral responses to demands that individuals perceive as threats to their wellbeing, whether from within or without them. To cope, one must attempt to lessen the threat of harm, loss, or grief that is frequently related to such situations. People experience stress anytime they confront circumstances that are more difficult for them to handle (Lazarus and Folkman, 1984).

College students use various coping strategies to handle stressful circumstances, which depends on how they perceive their level of self-efficacy (Gárriz et al., 2015). Most of the coping literature identifies two primary coping mechanisms: emotion-focused and problem-focused coping mechanisms (Cho et al., 2021). Problem-focused coping involves altering the external environment (e.g., enlisting help from others to solve the issue) or the internal environment (e.g., cognitive restructuring). Emotion-focused coping refers to coping mechanisms used to control intense emotions such as substance abuse and emotional venting. Contrarily, Algorani and Gupta (2023) included meaning-focused and social coping, also referred to as support-seeking coping strategies. An individual derives meaning from the conflicting situation while using meaning-focused coping and seeks emotional or instrumental support from social networks while using social coping. Later, Meyerson et al. (2022) mentioned a fifth coping strategy known as avoidance-focused coping.

College students face academic stress and need to be equipped to handle it. Notably, FGCS reported being more stressed than CGCS because they value different things in their educational experiences (Holt, 2022). The potential reasons include their difficulty in comprehending the elements of higher education, its environment, and the learning process. Some quit college early, and others cope with stressors from academic, personal, and work lives. The above literature led to the assumption that FGCS, as compared to CGCS, use less effective coping strategies in the first year of higher education.

The existing literature shows scant studies on the association between academic readiness and coping strategies. It is noted that there are more studies on stress among FGCS and its correlates or outcomes than stress coping strategies. de la Fuente et al. (2020) reported that college students with higher academic readiness used more effective coping strategies to regulate emotions and solve problems, which increased their academic performance. Furthermore, they found that flexible and adaptive use of coping strategies led students to better stress management, and they more readily coped with academic challenges (de la Fuente et al., 2020). Ma and Shea (2021) found that FGCS are academically less ready than their peers and lack the knowledge to cope with challenges of higher education. Because of having a lack of readiness and poor coping strategies, they perceive more barriers related to personal, academic, and financial responsibilities. Khan et al. (2022) reported a high level of stress overload among FGCS than CGCS when they are unable to cope with academic pressures and demands. This stress overload decreased their ability to deal with out-of-college obligations (Khan et al., 2022).

According to a qualitative study conducted in rural China, FGCS struggled because their parents had little knowledge and experience of the higher education system. They also lacked concrete coping mechanisms to deal with the college transition and choosing their study major (Cheng et al., 2023). In another qualitative study, Watts et al. (2022) listed reduced academic readiness among a few salient barriers that FGCS face. However, FGCSs rely on some effective coping strategies, such as seeking support from peer networks and institutions, besides employing personal strategies such as time management. The lack of studies necessitates exploring the association between academic readiness and coping strategies among college students. It is hypothesized that a higher level of academic readiness will be associated with more effective coping strategies among college students.

In earlier research, gender is shown to have a substantial impact on college academic readiness and coping strategies. According to Ishitani (2003), girls are more likely to be FGCS than non-FGCS. They were 61% more likely than boys to leave school in their fourth year, and 57% of girls were more likely to do so in their third year due to a lack of academic readiness. Combs et al. (2010) found that boys and girls reported having significant differences in readiness scores in reading, math, and both subject areas. Another research finding stated that FGCS girls were more likely than FGCS boys to have academic and related problems and leave college without degree completion (Jenkins et al., 2013).

Several investigations discovered gender-based differences in the coping strategies of college students. Morgan (2014) assessed perceived stress and coping among 1,085 undergraduate psychology students (FGCS = 415 or 38%; CGCS = 665 or 61%) belonging to six ethnic groups. Findings showed that generation status was not a significant factor, and FGCS and CGCS had more similarities than differences concerning stress and coping strategies. Another study found significant gender differences in the prevalence of stress and the use of coping strategies among college students. They showed that girls experienced more stress because of using maladaptive coping strategies of self-blame and denial during college. They more frequently relied on instrumental and emotional support to cope with stress. At the same time, boys used more problem-focused strategies (Graves et al., 2021).

Because there is little research on how Pakistani FGCS cope with the demands of higher education in the absence of academic awareness and family support, the study aims to fill the gap in the literature on the issues FGCS face that hinder their educational success. Much literature is available on the problems, such as financial difficulties that cause them to drop out of school, low socioeconomic status, lack of confidence, peer pressure, and other factors. However, very few studies describe how these students deal with stressful situations in higher education. Moreover, little information is available on gender inequalities in terms of academic readiness and coping. The primary objective of this study is to determine the levels of academic readiness and its relationship with coping strategies among FGCS and CGCS in Pakistan. The gender differences between FGCSs and CGCSs were also examined in the present study. The following hypotheses were examined:

1. First-generation college students will have a lower academic readiness than continuing-generation college students at both Time 1 and Time 2.
2. First-generation college students will use less effective coping strategies than continuing-generation college students at both Time 1 and Time 2.
3. Higher academic readiness levels will be associated with more effective coping strategies among college students at both Time 1 and Time 2.
4. Girls’ college students will be more academically ready and use more effective coping strategies than boys’ college students at both Time 1 and Time 2.

## Methods

### Research design

The study uses a quantitative research design in which a cross-sequential method was adopted to examine the overtime relationship between academic readiness and coping strategies among two cohorts of FGCS and CGCS. Academic readiness was first examined when the undergraduate students entered the degree program and examined after 3 months because both variables are affected by other factors, and assessment more reliably counter-checks their link overtime.

### Participants

Data were collected from undergraduate students in three universities of Hazara division, KPK, Pakistan. This region has a low literacy level and FGCS outnumber CGCS in the target population. This is the reason that FGCS (*n* = 212) are about three times larger than CGCS (*n* = 88), who more plausibly belong to other regions and enrolled in these universities because of subject choices or their parents’ employment. Participants were purposively selected in both groups using inclusion and exclusion criteria. The parents of FGCS did not have a college degree, whereas at least one parent of CGCS had a four-year college degree. They were currently full-time undergraduate students in a college or university and were in the age range of 19–24 years (*M* = 21.5, *SD* = 3.41). It was necessary to have English language reading proficiency to self-report the questionnaires and be comfortable disclosing information about their generation status, parents’ education, and family’s socioeconomic background. The exclusion criteria pertained to unclear generation status or a student ambiguity in classifying oneself in either group. Researchers excluded part-time, graduate, or returning students, and those outside the specific age range. At both times, researchers approached 356 students, and discarded 56 questionnaires for missing and incomplete data. Among them, we used valid data from 300 students (boys = 190, girls = 110) for statistical analysis.

Several socio-demographic factors were included as control variables, namely gender, age, socioeconomic status (SES), and parental education level, that affect academic readiness and coping strategies among college students. Table 1 shows the number and percentages of the participants’ characteristics, highlighting potential differences between the SES backgrounds of families and parental education across FGCS and CGCS groups. Across both groups, boys represented a higher proportion than girls across the FGCS group (64.2%) and CGCS (61.4%) group. Overall, there were 63.3% boys and 36.7% girls in the total sample. Most of the students were aged 19–21 (78.7%), and 21.3% were between the ages of 22 and 24. The CGCS group had a slightly higher proportion of younger students, whereas the FGCS group had a higher proportion of older students. The majority of students belonged to middle SES, comprising 91.3% of the total participants. Only 21 students came from a high SES, and five students came from low SES backgrounds. None of the FGCS parents had university-level education, and a notable percentage of mothers (19.8%) and fathers (18.9%) were illiterate. Conversely, in the CGCS group, 70.5% of mothers and 74.5% of fathers had a university education. The education level of mothers was relatively lower for FGCS students. A higher proportion of FGCS mothers had primary and middle school education, and CGCS fathers had intermediate education. Controlling these variables allows for a more precise examination of the relationship between academic readiness, coping strategies, and students’ background characteristics.

**Table 1:** Demographic characteristics of participants (*n* = 300).

Demographic	FGCS (*n* = 212)		CGCS (*n* = 88)		Total (*n* = 300)	
	*n*	%	*n*	%	*n*	%
Gender						
Boys	136	64.2	54	61.4	190	63.3
Girls	76	35.8	34	38.6	110	36.7
Age						
19–21	162	76.4	74	84.1	236	78.7
22–24	50	23.6	14	15.9	53	21.3
SES						
Low	5	2.4	0	0	5	1.7
Medium	197	92.9	77	87.5	274	91.3
High	10	4.7	11	12.5	21	7.0
Mother education						
Illiterate	42	19.8	0	0	42	14
Primary	22	10.4	2	2.3	41	13.7
Middle	47	22.2	3	3.4	81	27
Matric	71	33.5	10	11.4	50	16.7
Intermediate	30	14.2	11	12.5	24	8
University	0	0	62	70.5	62	20.7
Father education						
Illiterate	40	18.9	1	1.1	41	13.7
Primary	11	5.2	2	2.3	13	4.3
Middle	34	16.0	1	1.1	35	11.7
Matric	55	25.9	5	5.7	60	20
Intermediate	72	34.0	10	11.4	82	27.3
University	0	0	69	78.4	69	23

These reflect the number and percentage of participants who answered “yes” to these questions.

## Measures

### Readiness and expectation questionnaire (REQ)

Jansen et al. (2013) developed the Readiness and Expectation Questionnaire (REQ) to assess various aspects of first-year students’ perceptions of their expectations and preparedness. We used only the readiness part of this measure, which comprises 20 items across four subscales: information processing (5 items), collaborative learning (6 items), time management (5 items), and readiness writing (4 items). The items are responded on a five-point Likert scale from 1 (*not sure*) to 5 (*reasonably sure*). The total score ranges between 20 and 100, with a higher score indicating better academic readiness. Example items include “I find it easy to listen and make notes at the same time,” and “I am confident in discussing in small groups.” The Cronbach’s alpha ranged from 0.56 to 0.86 among cross-cultural participants (Jansen et al., 2013).

### Coping scale

Hamby et al. (2015) developed the Coping Scale, which was partially adapted from previous instruments developed by Holahan and Moos (1987) and Spitzberg and Cupach (2008) to measure cognitive, emotional, and behavioral coping strategies. This scale has 13 items that are answered on a 4-point rating scale, labeled as 1 (*not true about me*), 2 (*little true about me*), 3 (*somewhat true about me*), and 4 (*mostly true about me*). The total score is the sum of all the items and ranges from 13 to 52. A higher score indicates better coping ability. Example items are “When dealing with a problem, I try to see the positive side of the situation” and “When dealing with a problem, I make compromises.” The Cronbach’s alpha of the Coping Scale ranged between 0.88 and 0.90 during pilot testing and the main study, respectively (Hamby et al., 2015).

### Procedure

After seeking approval from the Ethical Review Committee of the University of XYZ, three universities from Hazara Division KPK were approached for permission to collect data. As our study design was based on the cross-sequential method, the participants must partake twice. They were approached at the start of the semester and followed up 3 months later. Both questionnaires were directly administered to the participants. The students were asked to complete demographic information about gender, age, socioeconomic status, and parents’ education. They took 20 min, on average, to complete the questionnaires. The participants were ensured that their information would be kept confidential and used only for research purposes. At Time 2, a significant data loss happened due to participants’ mortality.

### Data analysis

The data were entered in SPSS version 26. Data screening was done before statistical analysis. Data normality was tested via skewness and Kurtosis. Cronbach’s alpha reliability of the scales was estimated. The study examined the relationship between academic readiness levels and coping strategies of FGCS and CGCS at Time 1 and Time 2. Two separate structural equation models were tested for two cohorts of college students, treating academic readiness as a predictor of coping strategies. The cross-lagged paths were not assessed because of missing data and unequal group size. The mean scores, standard deviations, percentages, and correlation coefficients were also computed. Lastly, independent sample *t*-tests were run to examine the differences between FGCS and CGCS and boys and girls in college students’ academic readiness and coping strategies.

### Results

Findings show that the alpha reliability coefficient of the Readiness and Expectation Questionnaire was 0.84, and the Coping Scale was 0.70. The response rate was 74.07%. Table 2 provides information about the descriptive characteristics of the academic readiness scale and coping strategies for FGCS and CGCS at T1 and T2. Results show an increase in the mean scores of FGCS, CGCS, and the overall sample on readiness and coping strategies from Time 1 to Time 2. The only exception is the CGCS mean score on REQ, which declined by a few decimal points from Time 1 to Time 2. Participants’ overall mean REQ score increased from Time 1 (M = 70.92, SD = 14.50) to Time 2 (M = 73.29, SD = 14.77). Likewise, the overall mean CS score increased from Time 1 (M = 35.08, SD = 6.10) to Time 2 (M = 37.11, SD = 5.34). At Time 1 and 2, the minimum REQ score for FGCS and the total sample was 36. In contrast, for the CGCS cohort, it was 44 at Time 1 and 40 at Time 2. The mean CS score for CGCS ranged from 20 to 49 at Time 1 and 26 to 49 at Time 2. The minimum score was between 20 and 23, but there was less variation in the maximum CS score across groups and overtime.

**Table 2:** Descriptive statistics for academic readiness and coping strategies (*n* = 300).

Groups	Scales	T1					T2				
		Range					Range				
		*M* (*SD*)	Min	Max	Skew	Kurt	*M* (*SD*)	Min	Max	Skew	Kurt
FGCS	REQ	68.86 (14.08)	36	100	0.08	−0.96	72.58 (14.34)	36	100	−0.03	−1.00
	CS	34.43 (6.12)	20	49	−0.06	−0.72	36.77 (5.40)	26	49	−0.09	−0.76
CGCS	REQ	75.87 (14.37)	44	100	−0.19	−1.03	75.00 (15.70)	40	100	−0.35	−1.04
	CS	36.63 (5.85)	23	49	−0.03	−0.44	37.93 (5.13)	23	48	−0.21	−0.35
Total	REQ	70.92 (14.50)	36	100	0.02	−0.99	73.29 (14.77)	36	100	−0.13	1.04
	CS	35.08 (6.10)	20	48	−0.06	−0.59	37.11 (5.34)	23	49	−0.13	0.67

REQ, Readiness and Expectation Questionnaire; CS, Coping Scale; M, Mean; SD, Standard deviation; Kurt, Kurtosis.

The skewness value of academic readiness indicates a nearly symmetrical distribution in the FGCS cohort and a slightly negatively skewed distribution in the CGCS cohort (−0.19). In contrast, its Kurtosis is somewhat platykurtic and slightly leptokurtic in the first and second cohorts. The skewness value of coping strategies indicates a symmetrical distribution, while its Kurtosis value shows a flat peak for both cohorts.

**Figure 1:**
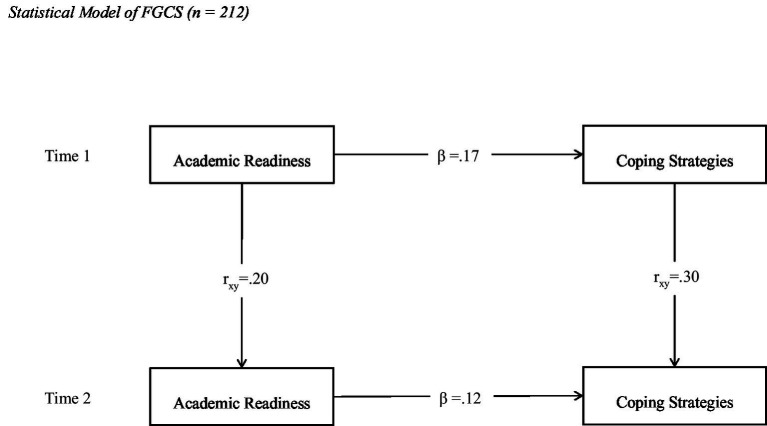
Statistical model of FGCS (*n* = 212).

Figure 1 demonstrates the relationship between academic readiness and coping strategies for FGCS. The beta value of 0.17 at Time 1 and 0.12 at Time 2 suggests that academic readiness positively predicts coping strategies, but this association is weak. The autoregressive paths show that academic readiness at Time 1 is positively associated with academic readiness at Time 2 (*r* = 0.20). Similarly, coping strategies at Time 1 are positively associated with coping strategies at Time 2 (*r* = 0.30). The overtime stability of the autoregressive paths indicate that FGCS with higher academic readiness at baseline reported having effective coping strategies. The fit indices showed acceptable model fit with the data [*χ*^2^ (3) = 19.74, *p* = 0.00; CFI = 0.95; RMSEA = 0.04; SRMR = 0.06].

**Figure 2:**
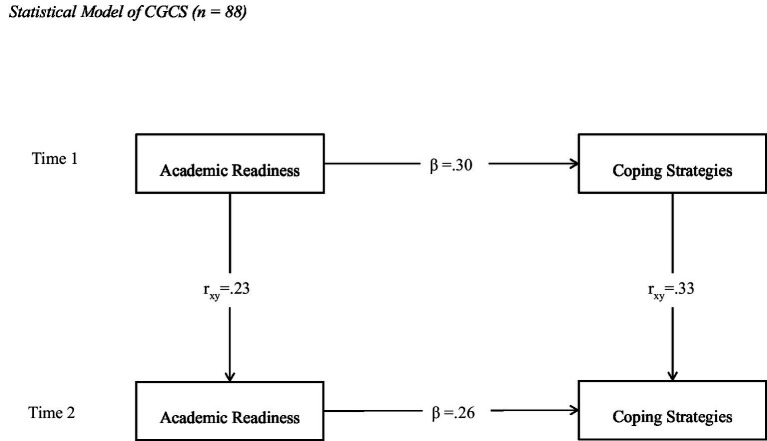
Statistical model of CGCS (*n* = 88).

Figure 2 demonstrates the relationship between academic readiness and coping strategies for CGCS. Findings show that academic readiness positively predicted coping strategies at Time 1 (ß = 0.30) and Time 2 (ß = 0.26). The autoregressive paths between Time 1 and Time 2 exhibit stability of academic readiness (*r* = 0.23) and coping strategies (*r* = 0.33). The model fit was in the acceptable range [*χ*^2^ (3) = 16.41, *p* = 0.00; CFI = 0.95; RMSEA = 0.05; SRMR = 0.06].

Table 3 exhibits the results of the independent samples t-test. It is hypothesized that FGCS will have lower academic readiness and less effective coping strategies than CGCS at Times 1 and 2. FGCS had lower mean scores on academic readiness and coping strategies than CGCS at Time 1. Nevertheless their scores increased at Time 2, leading to non-significant mean group differences on REQ [*t*(298) = −3.87, *p* < 0.001] and CS [*t*(298) = −1.30, *p* = 0.20]. Thus, the findings partially support the hypotheses with the presence of significant differences in the academic readiness and coping strategies of both cohorts at Time 1 only.

**Table 3:** Differences between FGCS and CGCS on academic readiness and coping strategies (*n* = 300).

Time	T1							T2						
Groups	FGCS (*n* = 212)	CGCS (*n* = 88)	*t*	*p*	LL	UL	Cohen’s *d*	FGCS (*n* = 212)	*CGCS* (*n = 88*)	*t*	*p*	LL	UL	Cohen’s *d*
Variables	*M* (*SD*)	*M* (*SD*)						*M* (*SD*)	*M* (*SD*)					
Ready	68.86 (14.54)	75.86 (14.45)	−3.87*	0.00	10.55	−3.44	0.48	75.85 (14.37)	75.00 (15.70)	−1.30	0.20	6.11	1.26	0.17
Coping	34.43 (6.12)	36.63 (5.85)	−2.86*	0.00	0.37	−0.68	0.37	36.77 (5.40)	37.93 (5.13)	−1.72	0.09	2.49	0.17	0.22

M, Mean; SD, Standard deviation; LL, Lower limits; UL, Upper limit. *p < 0.05.

Table 4 presents gender differences between FGCS and CGCS on the readiness and coping scales at two times. Results show non-significant differences at Time 1 and Time 2. The exceptions are FGCS girls (M = 70.28, SD = 14.00) with higher academic readiness scores than boys (M = 66.33, SD = 13.96) at Time 1. CGCS girls (M = 38.40, SD = 5.46) significantly outscored boys (M = 33.88, SD = 5.42) on average coping scores at Time 1. At Time 2, there were non-significant gender differences in academic readiness and coping strategies scores between FGCS and CGCS. Cohen’s d values indicate small effect sizes ranging from 0.09 to 0.41. The readiness and coping scores showed significant differences between girls and boys at Time 1 for the overall sample, where girls had higher scores than boys. These differences were (*t* (300) = 2.50, *p* = 0.01, Cohen’s *d* = 0.31) and (*t* (300) = 2.47, *p* = 0.01, Cohen’s d = 0.32), respectively. These differences suggest that gender plays a role in shaping readiness and coping strategies among college students.

**Table 4:** Gender differences between FGCS and CGCS on academic readiness and coping strategies (*n* = 300).

Groups	Time	T1							T2					
		Variables	*M (SD)*	*T*	*p*	LL	UL	Cohen’s d	*M (SD)*	*T*	*p*	LL	UL	Cohen’s d
FGCS (*n* = 212)	Girls (*n* = 136)	Ready	70.28 (14.00)	1.97*	0.05	0.00	7.90	0.28	73.72 (14.04)	1.56	0.12	−0.84	7.23	0.23
	Boys (*n* = 76)		66.33 (13.96)						70.53 (14.73)					
	Girls	Coping	34.70 (6.60)	0.84	0.40	−0.99	2.47	0.13	36.50 (5.45)	−0.99	0.33	−2.29	0.76	0.14
	Boys		33.96 (5.15)						37.26 (5.31)					
CGCS (*n* = 88)	Girls (*n* = 53)	Ready	78.15 (14.64)	1.87	0.06	−0.37	12.08	0.41	75.54 (15.49)	0.40	0.69	−5.48	8.26	0.09
	Boys (*n* = 35)		72.29 (13.62)						74.15 (16.23)					
	Girls	Coping	38.40 (5.46)	3.77*	0.00	2.14	6.89	0.83	37.63 (4.94)	−0.68	0.49	−3.02	1.46	0.16
	Boys		33.88 (5.42)						38.41 (5.47)					
Total (*n* = 300)	Girls (*n* = 190)	Ready	72.49 (14.58)	2.50*	0.01	0.92	7.71	0.31	74.24 (14.45)	1.46	0.14	−0.88	6.06	0.18
	Boys (*n* = 110)		68.17 (14.07)						71.65 (15.25)					
	Girls	Coping	35.74 (6.50)	2.47*	0.01	0.00	0.37	0.32	36.82 (5.32)	−1.25	0.21	−2.05	0.46	0.16
	Boys		33.94 (5.21)						37.62 (5.36)					

M, Mean; SD, Standard deviation; LL, lower limits; UL, upper limit. *p < 0.05.

## Discussion

The present research primarily aimed to compare and examine the role of academic readiness in predicting coping strategies among FGCS and CGCS at two different time points. Our study involved testing two cross-sequential models to determine if academic readiness predicts coping strategies among college students. Both models demonstrate a small but significant positive association between both variables at Time 1 and Time 2. The autoregressive paths of both constructs show stability—the academic readiness at Time 1 positively predicted academic readiness at Time 2. Similarly, coping strategies at Time 1 positively predicted coping strategies at Time 2. We tested four hypotheses while comparing FGCS with CGCS at both times.

### Academic readiness differences between FGCS and CGCS

The first hypothesis assumes that FGCS will have a lower academic readiness level than CGCS at Time 1 and Time 2. Findings show that the academic readiness of FGCS, on average, was lower than that of CGCS at the beginning of college, and they were less prepared to enter college. The CGCSs were more college-ready and had more effective coping strategies at Time 1. In Time 2, the average scores of FGCS on the measures of academic readiness increased to be equal to those of the CGCS cohort and presented non-significant differences. This suggests that FGCS may adapt overtime, potentially narrowing the readiness gap between these groups. Thus, the findings partially support the first hypothesis, given the significant difference between both groups only at Time 1. This finding aligns with LeBouef and Dworkin (2021) who state that FGCS often arrive at college academically less prepared than their peers, and they may fall behind at the beginning of their college careers because of a lack of family support and resources. The increase in readiness also indicates that students develop greater confidence and preparedness as they progress in their academic journey.

### Coping strategy differences between FGCS and CGCS

FGCSs are the first in their family to enter college and feel more challenged to achieve academic success than CGCSs. Because of being less prepared for higher education, they find it difficult to cope with the everyday challenges of college life. According to the second hypothesis, FGCS use less effective coping strategies than CGCS at both Time 1 and Time 2. Findings showed that the average scores of FGCS were less than those of CGCS on the measure of coping strategies at Time 1. These differences disappeared at Time 2; both cohorts had equal use of coping strategies. These findings provide partial support to the second hypothesis and support Ishitani (2003) study, which found that FGCS students are more likely than CGCS never to finish their degrees and require longer due to poor coping strategies. Another study found that FGCS suffer more than CGCS because they lack effective coping methods for dealing with the move to college (Cheng et al., 2023).

### Association between academic readiness and coping strategies

First-year students transition from high school to college under-prepared or unprepared. However, they must adapt to the new environment for academic success. The existing literature led to the assumption that academic readiness will positively influence coping strategies in a way that the higher level of readiness will be associated with more effective coping strategies among college students at both Time 1 and Time 2. The rationale behind this assumption was that academically ready students do not feel challenged on tests of their ability and are better able to handle problems than academically less prepared. The academic readiness was treated as a predictor of coping strategies among college students. The findings of cross-sequential models support that academic readiness influences coping strategies in a positive direction among FGCS and CGCS, and this association remains stable overtime. However, the magnitude of these paths was small, as shown in Figures 1, 2. These findings support this hypothesis, as academic readiness positively predicted coping strategies at both time points for both FGCS and CGCS.

Another interesting observation is that the magnitude of these associations was almost double for CGCS than FCGS. In the FGCS model, the beta coefficients between readiness and coping were 0.17 at Time 1 and 0.12 at Time 2. In contrast, the beta coefficients in the CGCS model were 0.30 and 0.26 at Time 1 and Time 2. It is inferred from these findings that FGCSs had a lower level of academic readiness and less effective coping strategies than CGCSs at both Time 1 and Time 2. These findings support the hypothesis and align with Antonelli et al. (2020) and Crowell (2023), who found that FGCS have lower college readiness and retention than CGCS. House et al. (2020) reported that FGCS face more stressors than non-FGCS due to the absence of family support and friends support, which lowers their coping skills.

### Gender differences in academic readiness and coping strategies

A secondary aim of the present study was to investigate gender differences between FGCS and CGCS. It was hypothesized that girls would score higher on academic readiness and coping than boys. The overall finding presented in Table 4 shows partial support for this hypothesis. Girls scored higher than boys on both study variables at Time 1. Specifically, academic readiness was higher among FCGS girls, and coping was higher among CGCS girls than their counterparts at Time 1 only. However, these gender differences were observed at Time 2, where no significant differences were observed in either academic readiness or coping strategies. All t-values were non-significant and had small effect sizes. Despite having negligible differences, the analysis of mean scores reveals some fascinating information. These findings imply that FCGS become more resilient overtime and use effective coping strategies as CGCS do, nullifying their group differences at Time 2. Moreover, girls are more prepared than boys when entering college. Irrespective of being FGCS or CGCS, girls had relatively higher mean scores on the readiness scale and coping strategies than boys at both Time 1. However, boys surpassed girls in coping strategies at Time 2. The overall gender differences also favor boys for higher scores on coping than girls. This finding implies that boys use more effective coping mechanisms to overcome academic issues.

## Limitations and suggestions

This section highlights some prominent challenges, creating opportunities for future research in this field. The study solely focused on undergraduate students, which restricts the applicability of the findings to graduate students. The reliance on self-reported data introduces social desirability and potentially biased representation of participants’ academic readiness and coping strategies. Another limitation pertains to the restricted sample, which was limited to three universities in the Hazara division, KPK, Pakistan. Consequently, the results may have limited generalizability to other regions of Pakistan. To address these limitations, future research should include undergraduate and graduate students and a larger diversified sample from various areas across Pakistan to enhance the generalizability of the findings. Researchers should employ objective measures to minimize the potential influence of social desirability bias and consider adopting a mixed-methods approach to gain a more comprehensive understanding of the academic readiness and coping strategies of FGCS and CGCS. Moreover, future research should bridge the gaps between FGCS and CGCS, exploring the impact of interventions, preparatory programs, and mentorship on their academic readiness and coping strategies.

## Implications

The study pinpoints how crucial it is to provide first-year FGCS with an awareness of higher education to make them college-ready. The findings hold theoretical and practical implications. Theoretical implications stem from its contribution to the existing literature on coping mechanisms among FGCS in Pakistan and identify factors influencing their academic preparedness. The findings highlight that FGCS, particularly men, initially face unique challenges in adapting to the demands of higher education and overcome them with time. Additionally, the role of gender in shaping academic readiness and coping strategies warrants further investigation, particularly in understanding why initial differences may diminish overtime.

From a practical standpoint, it indicates a need for diagnostic screening of specific problems among first-year college students in higher education institutions. The institution should evaluate first-year students’ academic readiness and coping strategies and the opportunities for adjustment to FGCS. Those needing support services should receive prevention and intervention during the transition to higher education. The study’s findings help cater to the specific needs of college students and thus promote their academic success. Educators and college administrators make arrangements, such as preparatory programs, mentorship, financial assistance, and collaboration with family/parents to protect the students’ wellbeing. The providers of counseling services and policymakers can design various support services to enhance academic readiness, manage stress, and use adaptive coping strategies, particularly at the beginning of college. The cross-sequential design, sample group, i.e., undergraduate students, and data collection time, i.e., using two cohorts for comparison, strengthen the study’s implications.

## Conclusion

This study aimed to fill a knowledge gap regarding the coping mechanisms of first-generation college students (FGCS) in Pakistan who lacked parental academic awareness and support. The results show that FGCS encounter unique difficulties when adjusting to the demands of higher education because their parents need to provide them with academic guidance. FGCS had low readiness and less effective coping on average than CGCS at the beginning of the academic year. They adapted to the educational environment and developed better coping mechanisms more quickly, and the difference between FGCS and CGCS disappeared. Initially, FGCS were less ready for higher education but they improved overtime and there was a less significant difference between both cohorts in scores for academic readiness. Girls were more ready and better at coping than boys, irrespective of their generation status. The cross-sequential design, sample group selection, and duration of data collection all contribute to the study’s strength. Educators can customize interventions and support services to address difficulties of FGCS’ in navigating higher education and fostering their personal and academic growth.

## Data Availability

The raw data supporting the conclusions of this article will be made available by the authors, without undue reservation.
